# Globefish‐Inspired Balloon Catheter with Intelligent Microneedle Coating for Endovascular Drug Delivery

**DOI:** 10.1002/advs.202204497

**Published:** 2022-10-18

**Authors:** Xiaoxuan Zhang, Yi Cheng, Rui Liu, Yuanjin Zhao

**Affiliations:** ^1^ Department of Rheumatology and Immunology Nanjing Drum Tower Hospital School of Biological Science and Medical Engineering Southeast University Nanjing 210096 China; ^2^ Oujiang Laboratory (Zhejiang Lab for Regenerative Medicine, Vision and Brain Health) Wenzhou Institute University of Chinese Academy of Sciences Wenzhou 325001 China; ^3^ Chemistry and Biomedicine Innovation Center Nanjing University Nanjing 210023 China

**Keywords:** balloon catheter, bioinspired, drug delivery, microneedles, Trumprestenosis

## Abstract

Balloon catheters exhibit important values in treating cardiovascular diseases, while their functions are still under improvements. Here, inspired by the thorn‐hiding and deflating–inflating characteristics of globefish, intelligent balloon catheters decorated with invisible microneedles are presented for endovascular drug delivery to inhibit postintervention restenosis (PIRS). These microneedle balloon catheters (MNBCs) fabricated by dipping and rolling‐assisted template replication contain three coating layers of sandwiched drug‐carrying microneedles and black phosphorus (BP)‐carrying gelatin. During the emplacement, the microneedles of MNBCs are hidden under the outermost gelatin protective layer, allowing smooth movements inside the blood vessel. After reaching the destination, the embedded BP converts near infrared (NIR) into heat, increases local temperature, and melts the gelatin layer, enabling the exposure and vascular penetration of the microneedles. Besides, as the innermost gelatin also melts, the microneedles can detach from the balloon catheter and be left inside the blood vessel for continuous drug release. Based on advantages of responsiveness, penetration capacity, and biosafety, it is demonstrated that the MNBCs behave satisfactorily in delivering rapamycin to inhibit abdominal aorta restenosis in rats. All these features indicate that these MNBCs are promising medical devices for clinical applications.

## Graphical abstract

Inspired by changeable thorny appearances and inflatable behaviors of globefish, intelligent hierarchical balloon catheters with concealed microneedles are fabricated for treating cardiovascular disorders. These balloon catheters contain drug‐carrying microneedles between photothermal‐responsive gelatin layers. The pointed microneedles are hidden during placement, while they are exposed and left inside tissues under near infrared (NIR) for responsive and continuous endovascular drug delivery.

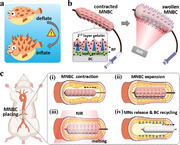

## 1 Introduction

Postintervention restenosis (PIRS) has a high morbidity rate and negatively impairs the therapeutic effects of interventional operations such as angioplasty and stent implantation, posing a great threat to human health.^[^ ^1^, ^2^ ^]^ The main characters of PIRS include phenotypic transformation, excessive proliferation, and migration of vascular smooth muscle cells, and their induced intima thickening and lumen stenosis.^[^ ^3^ ^]^ To prevent restenosis, oral administration of anti‐platelet coagulation, lipid‐lowering drugs such as aspirin and simvastatin is employed in clinic.^[^ ^4^ ^]^ Whereas, oral drug delivery faces shortcomings including low utilization ratio, obvious side effects, and disability to realize targeted and in situ administration.^[^ ^5^, ^6^, ^7^, ^8^, ^9^ ^]^ As an alternative, drug eluting balloon catheters coated with rapamycin, paclitaxel, or other drugs that can inhibit vascular smooth muscle cell proliferation are developed,^[^ ^10^, ^11^, ^12^, ^13^, ^14^, ^15^ ^]^ which are placed inside the artery and will stay at the lesion site for several minutes to achieve local and precise delivery.^[^ ^16^ ^]^ Although with some improvements, drugs are simply sprayed on these balloon catheters, leading to an unstable connection and thus inevitable drug loss during balloon emplacement. Besides, due to the limited administration time and the physiological barriers of the intima, drug absorption rate and therapeutic effect of the traditional drug eluting balloons are far from satisfaction. Therefore, new stratagems for local endovascular drug delivery to prevent restenosis are still highly anticipated.

**Figure 1:**
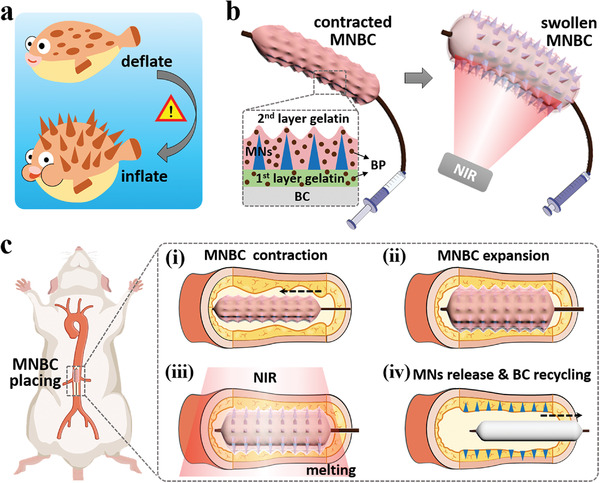
Schematics of globefish‐inspired MNBCs and their applications. a) Globefish generally deflate and hide their thorns, while they inflate and exhibit their thorns in danger. b) MNBCs contain three coatings: two BP‐encapsulated gelatin layers and microneedles (MNs) in between. MNBCs can not only contract and conceal MNs, but also swell and expose MNs under NIR irradiation. c) Processes of placing the MNBC inside the rat abdominal aorta: i) contracting the MNBC for successful passing; ii) expanding the MNBC for full contact; iii) applying NIR for melting gelatin layers; iv) leaving microneedles in tissue and recycling the bare balloon catheter (BC).

Herein, inspired by the structure and responsiveness of globefish, we present novel intelligent balloon catheters coated by invisible microneedles with desired features to deliver drugs endovascularly for PIRS prevention, as schemed in **Figure** **1**. Globefish is a kind of creature that hides their thorns in normal situations. When they sense danger, the globefish would inhale water or air, make their chest and abdomen swell, as well as expose and erect their thorns for self‐protection. Such a fantastic thorn‐hiding and deflating–inflating feature has aroused great interest among biologists, structuralists, and mechanists.^[^ ^17^, ^18^, ^19^, ^20^, ^21^ ^]^ Coincidentally, balloon catheters happen to deflate during movement inside the blood vessels, and inflate when reaching targeted sites.^[^ ^22^ ^]^ Thus, it is conceivable that the coupling of globefish structures and drug‐loading balloon catheters would form a valuable and unprecedented endovascular drug delivery system with high efficiency and responsiveness. So far as we know, the practical applications of globefish‐inspired devices in biomedical sciences have seldom been explored, and their combination with balloon catheters is almost unheard of.^[^ ^23^, ^24^ ^]^

In this paper, we integrate the globefish‐inspired invisible thorn‐shaped structures as well as responsive deflating–inflating function with medical balloon catheters by using dipping and rolling‐assisted template replication approaches for endovascular drug delivery. Different from traditional skin‐applied microneedle devices,^[^ ^25^, ^26^, ^27^, ^28^, ^29^ ^]^ such microneedle balloon catheters (MNBCs) were with special three‐layered coating in which drug‐carrying microneedles were sandwiched between black phosphorus (BP)‐carrying gelatin, thus they were hiding their needles during emplacement while revealing them at designated locations. During this process, the MNBCs could smoothly enter the arteries and reach the site of diseases with the protection of the outermost gelatin shell. As the photothermal‐convertible BP^[^ ^30^, ^31^, ^32^, ^33^, ^34^, ^35^ ^]^ was added in the gelatin, the surface temperature of the inflated MNBCs would increase under near infrared (NIR) irradiation, which could melt the gelatin shell and expose the microneedles, and thus enabled endangium penetration. Besides, the innermost gelatin also melted, realizing the separation between the retentive microneedles and the balloon catheter, and achieving the sustainable and long‐term drug release after the balloon catheter was removed. Based on these features, the practical values of MNBCs were further proved by inhibiting abdominal aorta restenosis in rats, indicating that they may provide new therapeutic ideas for PIRS and other cardiovascular disorders.

## 2 Results and Discussions

**Figure 2:**
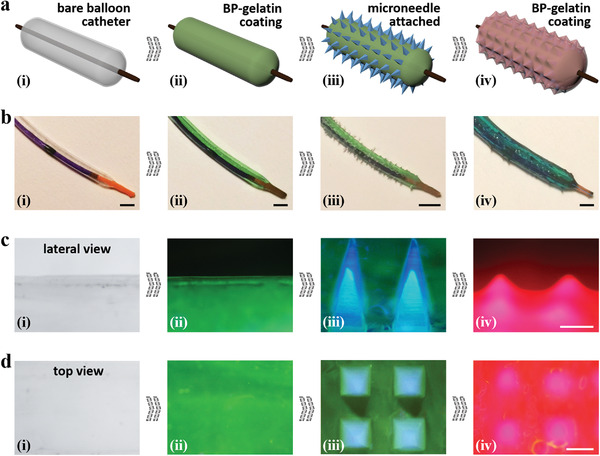
Schematics and characterization of the fabrication process of MNBCs. a) Schematics showing the integration of different coating layers on the balloon catheter: i) bare balloon catheter; ii) adding the BP‐containing gelatin coating; iii) attaching the microneedle coating; iv) adding another BP‐containing gelatin coating. b) Macrographs of different steps of the integration. The gelatin layers were dyed green. c) Micrographs of the integration steps from lateral views. d) Micrographs of the integration steps from top views. In (c, d), the inner gelatin layer, microneedles, and the outer gelatin layer were dyed green fluorescence, blue fluorescence, and red fluorescence, respectively. Scale bars: 2 mm in (b) and 250 µm in (c, d).

In a typical experiment, the three coating layers were integrated on the bare balloon catheter one after another, as shown in **Figure** **2**a,b. Specifically, the gelatin layers were added by immersing the balloon catheter in the heated gelatin solution, and then taking it out at room temperature for solidification; while the microneedles were attached by firstly filling *N*‐acryloyl glycinamide (NAGA) pregel into a template lined with cavities, placing the balloon catheter on the template, selectively curing the pregels that directly contacted the catheter, and finally rolling the catheter and repeating the curing until complete coating (Figure S1, Supporting Information). To clearly distinguish the different coating layers, green fluorescence, blue fluorescence, and red fluorescence were employed to stain the inner gelatin layer, the microneedles, and the outer gelatin layer, respectively (Figure 2c,d). It was observed that the inner gelatin layer wrapped the surface of the balloon uniformly and fit with the microneedles well, and that the outer gelatin layer covered majority parts of the microneedles, making the MNBCs relatively smooth. The dimension of the microneedles could be adjusted by using different templates. Here their height and side length were 550 and 250 µm for display. Notably, due to the deformability and flexibility of gelatin and NAGA materials, these coating layers showed satisfactory conformity as the balloon catheter inflated or contracted (Figure S2, Supporting Information).

**Figure 3:**
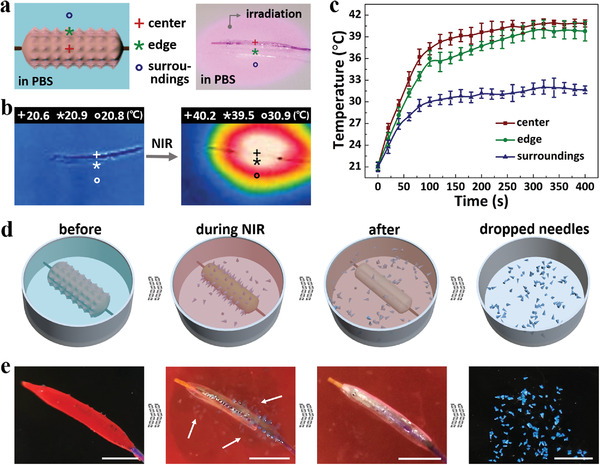
NIR‐responsive heating and separation. a) Schematic and macrograph showing the temperature measuring points. b) Infrared thermal images of the MNBC before and after NIR irradiation. c) Temperature changing profiles of the central position, edge, and surrounding environment of MNBCs (*n* = 4 for each group). d) Schematics showing the melting of gelatin coatings and the separation of microneedles from the MNBC under NIR irradiation. e) Corresponding macrographs showing the melting and separation processes. The inner gelatin layer, microneedles, and the outer gelatin layer were dyed green, blue, and red, respectively. Scale bars: 5 mm.

By mixing BP nanosheets into gelatin, these gelatin layers were imparted with the ability to respond to NIR ra. It was found that under NIR irradiation, the central part, edge, as well as surrounding environment of the MNBC were all heated up to different degrees, as shown in the infrared thermal images of **Figure** **3**a,b. For detailed analysis, the temperature changes of such three parts over time were recorded and plotted (Figure 3c). Results showed that all the temperature rose up to their separate plateaus in about 100 s. Besides, the temperature increasing profiles of the central part and the edge were similar, while the heating of the surrounding environment was comparatively indistinctive, which demonstrated that the MNBCs would not burn the adjacent tissues and ensured their safety during practical applications. In addition, to better adjust the photothermal conversion properties of MNBCs, the parameters that might influence the temperature increasing profiles were investigated. The BP concentrations were found to have only slight influence on the maximum temperature (Figure S3a, Supporting Information). On the contrary, the NIR power could affect the heating process significantly (Figure S3b, Supporting Information). As the NIR power boosted, both the speed and extremum of the temperature of MNBCs significantly increased. Considering the melting temperature of gelatin, BP concentration of 0.1 mg mL^−1^ and NIR power of 5 W were chosen as an optimized condition.

Benefitting from the photothermal conversion ability of BP and the sol–gel phase transition characteristics of gelatin, the microneedles could be exposed and detached from the balloon catheter under NIR irradiation. For illustration, the MNBC was immersed in phosphate buffer saline (PBS) buffer solution and the NIR emitter was applied from above (Figure S4, Supporting Information). It could be seen that as the temperature gradually went up, both the outer gelatin layer and the inner gelatin layer melted, leading to the complete separation of microneedles, as shown in Figure 3d,e. In addition, since the microneedles should actually penetrate the blood vessel rather than only stay on the surface, their mechanical properties were studied. A force‐measuring system was first employed for quantitative evaluation. Data from this system showed that the microneedles could bear more than 40 mN per needle without fracture, indicating an acceptable mechanical strength (Figure S5, Supporting Information). Besides, forces required for blood vessel penetration were also detected by using a 10 × 10 microneedle array to pierce a fresh ex vivo pig aorta tissue and recording the force changes (Figure S6a, Supporting Information). The penetration force was found to be about 2.2 N for the entire array, which was weak and easy to achieve (Figure S6b, Supporting Information). The bright‐field and fluorescence images of the pig aorta tissue after microneedle application further demonstrated the successful penetration (Figure S7, Supporting Information). The degradation ability of the microneedles was further tested by immersing NAGA blocks in buffer solutions. The in vitro degradation rate was found to be about 25% after 7 days (Figure S8, Supporting Information).

**Figure 4:**
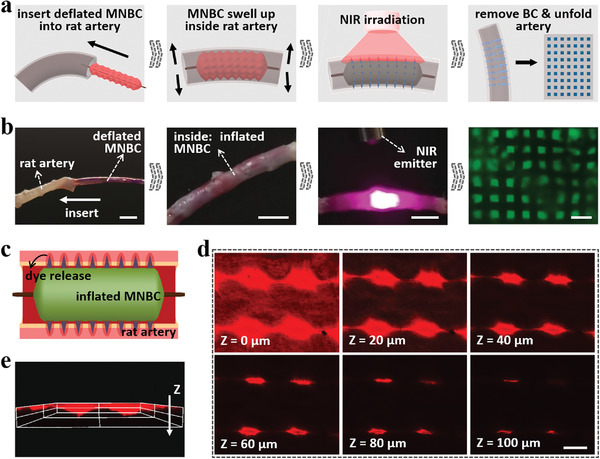
MNBC penetration and separation inside in vitro rat abdominal aorta. a) Schematics of inserting the deflated MNBC into the rat artery, inflating the MNBC, turning on NIR, removing bare balloon catheter, and unfolding the artery with microneedles left inside. b) Corresponding macrographs of the operation process and fluorescence image of the left microneedles. The gelatin layers were dyed red and microneedles were stained with green fluorescence. c) Schematic showing the dye release from the MNBC to the rat artery. d) Confocal laser scanning microscopy images of the dye diffusing in the artery tissue. The images differ in heights (*Z* axis). e) Corresponding 3D reconstruction image. Scale bars: 3 mm and 250 µm in (b), and 100 µm in (d).

To vividly simulate practical intravascular conditions, a rat abdominal aorta was sampled and prepared for MNBC application, as schemed in **Figure** **4**a. During this process, the MNBC deflated and shrank to ensure insertion into the abdominal aorta, and would inflate and swell to fully fill the blood vessel after insertion (Figure 4b). With NIR on, the gelatin layers melted inside the rat artery, and the deflated bare balloon catheter was then withdrawn from the artery. By unfolding the artery, the microneedles were found to be left in the tissue, which preliminarily proved the ideal in vivo performances of MNBCs. Besides, to find out the penetration and drug release depths, a fluorescent dye, Nile Red, was encapsulated in the microneedles (height: 100 µm) and the MNBC was placed inside the rat abdominal aorta with the same method (Figure 4c). Confocal fluorescence tomography and reconstruction images revealed that the drug permeation depth was more than 100 µm (Figure 4d,e).

**Figure 5:**
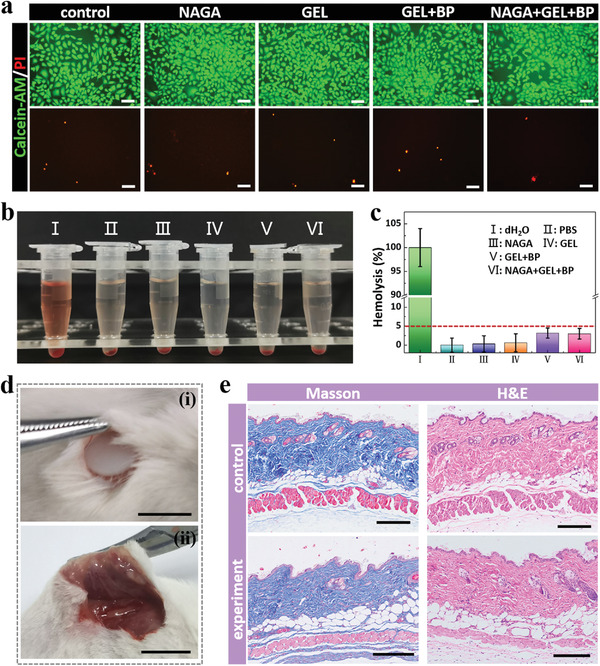
Biosafety and biocompatibility tests of MNBC coatings. a) The live/dead staining images of HUVECs in traditional culture medium (control) and leaching liquors of NAGA hydrogel (NAGA), gelatin hydrogel (GEL), BP‐containing gelatin hydrogel (GEL+BP), and BP‐containing NAGA‐gelatin mixed hydrogel (NAGA+GEL+BP) on day 3. b) Images of hemolysis test results. The groups are deionized water (I), PBS buffer solution (II), and leaching liquors of NAGA hydrogel (III), gelatin hydrogel (IV), BP‐containing gelatin hydrogel (V), and BP‐containing NAGA‐gelatin mixed hydrogel (VI). c) Corresponding hemolysis rates (*n* = 6 for each group). d) Macrographs of the subcutaneously implanted materials on i) day 0 and ii) day 28. e) Masson and H&E staining images of mouse skins without (control group) and with (experiment group) material implantation on day 28. Scale bars: 100 µm in (a), 1 cm in (d), and 200 µm in (e).

The biocompatibility of the coating layers of MNBCs should not be ignored. For this purpose, human umbilical vein endothelial cells (HUVECs) were cultured in the environments with coating layer materials immersing, and the cell viability was assessed. The live/dead staining images of HUVECs showed that all cells grew and proliferated well whether they were exposed to NAGA hydrogel, gelatin hydrogel, BP‐gelatin mixed hydrogel, BP‐NAGA‐gelatin mixed hydrogel or not (**Figure** **5**a). For more detailed quantification, CCK‐8 assay was also conducted and the amounts of viable cells were found to be similar in all the groups for consecutive three days, which further demonstrated the outstanding cyto‐compatibility of the coating layer materials (Figure S9, Supporting Information). In addition, as the MNBCs would directly contact blood during application, hemolysis tests of the coating layer materials were then executed by mixing fresh red blood cell buffer with material leaching liquors. Results showed that hemolysis rates of the coating layer materials were lower than 5%, indicating the satisfactory blood compatibility, as shown in Figure 5b,c.

In addition to cell‐relevant experiments, the coating layer materials of MNBCs were implanted under mouse skins to evaluate their in vivo safety. During this process, small incisions were created on the mouse dorsal skins for material placement (Figure 5d(i)). After the operation, the body weights of the mice with and without material implantation were recorded every four days. It was found that mice experiencing subcutaneous implantation and belonging to the control group both kept active and gained weight steadily, meaning that the coating layer materials had negligible negative impacts on the lives of the mice (Figure S10, Supporting Information). Besides, the coating layer materials degraded and lost intactness when the skins were sampled after 28 d, and no abnormal skin conditions were observed (Figure 5d(ii)). Also, the Hematoxylin–Eosin (H&E) and Masson staining images of the skin samples showed well‐defined skin layers, normal skin structures, and little inflammatory responses of the mice receiving material implantation (Figure 5e). Immumohistochemical staining of TNF‐*α* (an inflammatory cytokine) and CD68 (a biomarker for macrophages) further indicated that there was no obvious immune response around the implanted material (Figure S11, Supporting Information). Additionally, their major organs (heart, liver, spleen, lung, and kidney) all exhibited normal forms and structures, as seen in the H&E staining images of Figure S12 (Supporting Information). All the above results strongly illustrated that the MNBCs possessed desired biocompatibility and could be safely applied in vivo.

Before these MNBCs were actually used for endovascular delivery, their drug release profiles were investigated in vitro. Taking the lipophilicity and molecular weights of most clinically‐used PIRS drugs into account, the fluorescent oil‐soluble molecule Nile Red was chosen as the model drug and encapsulated inside NAGA microneedles. It was observed that the red fluorescent Nile Red was distributed uniformly inside the microneedle, as shown in the fluorescence images of Figure S13a (Supporting Information). By choosing NAGA hydrogels with different crosslinking degrees, drug release rates of the microneedles could be easily adjusted. In particular, the NAGA hydrogel would gradually degrade accompanied by the drug release and the loaded drugs were delivered almost completely in about 5 h, as plotted in Figure S13b (Supporting Information). Whereas, with the addition of the crosslinker, the NAGA hydrogel network became tighter and more stable, leading to the much lower release speed. Comprehensively considering the drug action time and drug bioavailability, the pure NAGA hydrogel was set as the microneedle material for following animal experiments. It should be mentioned that, to understand the drug release kinetics from the microneedles, we fit the drug release before plateau with Higuchi equation ($y\;=\;36.46\sqrt{x}+10.82,\;R^{2}\;\approx \;0.91$) and first‐order release equation (*y* = 89.85(1 − *e* ^−0.82x^), *R* ^2^ ≈ 0.99), respectively (Figure S14, Supporting Information). Results showed that the drug release could well fit the first‐order release equation.

**Figure 6:**
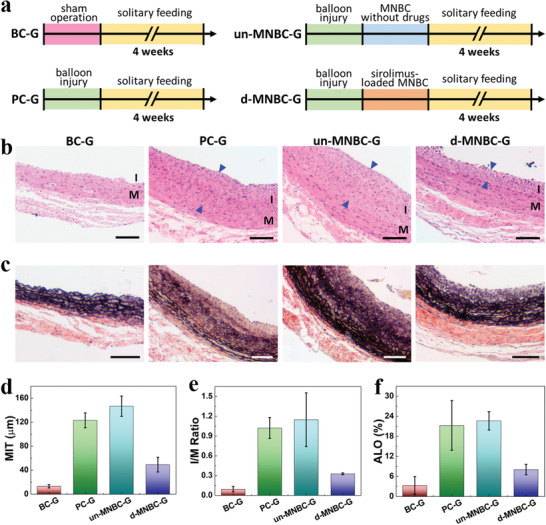
Timeline and results of treating the abdominal aorta restenosis rat models. a) Diagram of the experimental protocol of rat grouping, model establishment, and treatment. b) H&E staining of the abdominal aorta tissues from the four groups: BC‐G, PC‐G, un‐MNBC‐G, and d‐MNBC‐G, respectively. I and M indicate intima and media. The blue triangles indicate the intimal thickness. c) EVG staining of the abdominal aorta tissues from the four groups. d) Statistical analysis of the maximal intimal thickness (MIT) (*n* = 3 for each group). e) Statistical analysis of the intima to media area ratio (I/M ratio) (*n* = 3 for each group). f) Statistical analysis of the aortic luminal obliteration (ALO) (*n* = 3 for each group). Scale bars: 100 µm.

Aiming to test the practical therapeutic effects of MNBCs, balloon injury was employed to establish rat models of abdominal aorta restenosis, as shown in **Figure** **6**a. These rats were randomly divided into different groups: BC‐G as the blank control in which rats only experienced sham operation, PC‐G as the positive control in which rats received balloon injury but no treatment, un‐MNBC‐G in which the injured rats were treated by MNBCs without drug loading, and d‐MNBC‐G in which the injured rats were treated by rapamycin‐loaded MNBCs. It was worth noting that, depending on the dimension of medical balloon catheters, the diameter of MNBCs ranged from 2 to 6 mm, which determined the blood vessels that the MNBCs could be applied to. If too large, the MNBCs could not enter the blood vessels; if too small, they would not be able to fully contact the vascular endothelium. As for rat abdominal aortas, MNBCs with the diameter of 2 mm were suitable. Also, considering the thickness of rat abdominal aortas and the drug loading amount of MNBCs, the height and side length of the microneedles were both 100 µm. During the interventional operation, the abdominal aorta of the rat was exposed and an inflated bare balloon catheter passed through the intima to cause injury and to induce restenosis (Figure S15, Supporting Information). After the balloon catheter was withdrawn, the MNBC was applied and then exposed to NIR light for melting the gelatin layers, separating the microneedles, and guaranteeing drug release. The penetration depth of NIR‐I (700–1000 nm) to animal tissues ranged from ≈5 to ≈10 mm and varied for different organs/tissues,^[^ ^36^ ^]^ which would not influence the MNBC performances because the NIR only penetrated through a thin layer of blood vessel to reach the MNBCs.

To compare the pathological states of the rats, H&E staining was used at the abdominal aorta tissues 4 weeks postoperatively (Figure 6b and Figure S16, Supporting Information). As opposed to the clear vascular wall structure and normal intima morphology that was composed of a complete and continuous layer of endothelial cells in BC‐G, obvious aortic lesions were observed in PC‐G, demonstrating the successful establishment of the restenosis rat model. To be specific, vascular smooth muscle cells in PC‐G excessively proliferated and migrated to intima, leading to impaired vascular endothelium, thickened and elevated intima, and disordered three‐layer structure morphology. In stark contrast, intimal hyperplasia and lesion severity of d‐MNBC‐G were significantly reduced, preliminarily confirming the restenosis preventive effects of drug‐loaded MNBCs. Besides, destruction of elastic fibers was also a key pathological indicator, which could be marked by Verhoeff's Van Gieson (EVG) staining. Results showed that the structure and thickness of elastic fiber was relatively normal in d‐MNBC‐G compared to PC‐G (Figure 6c). Additionally, for quantitative analysis, the maximal intimal thickness, intima to media area ratio, and aortic luminal obliteration were calculated (Figure 6d–f). It was found that d‐MNBC‐G showed significantly lower values in the above three factors (49.32 ± 12.30 µm, 0.33 ± 0.01%, and 8.05 ± 1.57%, respectively) than PC‐G (123.17 ± 12.30 µm, 1.02 ± 0.16%, and 21.26 ± 7.42%, respectively). It should be mentioned that restenosis was the most severe in un‐MNBC‐G among all the groups, owing to the secondary damage caused by placing unloaded MNBCs. All these above features demonstrated the ideal endovascular delivery and disease curing capacity of the drug‐loaded MNBCs.

## 3 Conclusion

In summary, we got inspirations from the thorn‐covered skin and deflating–inflating body of globefish, and fabricated MNBCs that could hide their microneedles for endovascular drug delivery and PIRS inhibition. Such MNBCs were composed of three coating layers, which were BP‐carrying gelatin, drug‐carrying microneedles, and BP‐carrying gelatin from the interior to superficies. The gelatin layers were attached by dipping the balloon catheter in a heated gelatin solution; while the microneedle layer was added based on rolling the balloon catheter on a template and selectively solidifying the contacted pregel solutions. Because of the flexibility of hydrogels, these coating layers were well conformal to the balloon catheter and deformed with the inflating and deflating of the MNBC. Besides, due to protective effects of the outermost gelatin layer, the microneedles were concealed and the MNBC had a relatively smooth surface, making it easy to be placed inside the blood vessel. Additionally, attributed to the photothermal‐convertible ability of BP and the sol–gel transitional property of gelatin, the gelatin layers melted under NIR irradiation, exposing the microneedles and enabling vascular penetration. Notably, the inner gelatin layer also melted at this process, which separated the microneedles from the balloon catheter for keeping the microneedles inside the tissue and elongating the drug release time. It was demonstrated through intravascularly delivering rapamycin to treat abdominal aorta restenosis rat models that the application of MNBCs might be a practical clinical approach for cardiovascular therapy.

Compared to traditional balloon catheter devices, the most distinctive advantage of MNBCs is that they can effectively improve drug availability and drug absorption. Since drugs are loaded in the materials of MNBCs rather than sprayed on their surfaces, the MNBCs can reduce drug loss during the emplacement to a great extent. Also, benefitting from the separable microneedles with the ability to penetrate tissues, the MNBCs can overcome the intima barriers and prolong the drug delivery time. All these properties lead to improved pharmaceutical and therapeutic effects. In addition, the integration with NIR responsive materials further imparts the MNBCs with controllable drug release capacity and makes them more intelligent.

There is still a lot of room for the improvements of MNBCs. Firstly, the fabrication strategy can be further upgraded by developing automatic processing equipment. In this way, the parameters can be better controlled and the fabrication precision as well as reproducibility can be greatly enhanced. In addition to delivering rapamycin and similar small‐molecular drugs, future MNBCs can carry macromolecular peptide/protein drugs, gases, inorganic nanoparticles, liposomes, extracellular vesicles, viruses, and even cells for intravascular drug release. Besides, the hydrogel composition of the microneedles can be adjusted by adding some responsive components to allow for responsive drug release, controlled drug release, or staged release of multiple drugs. Additionally, magnetic responsiveness may also be introduced to MNBCs, and thus the MNBCs can be manipulated remotely and smartly inside the vessels. Moreover, the auxiliary parts of MNBCs can be further improved to solve the difficulty of applying bulky NIR generator to human patients. For example, ultrathin optical fibers can be assembled inside the balloon catheter with a NIR laser at the other end, which enables the self‐illumination of MNBCs and avoids the extra use of large NIR generators. These advancements are believed to facilitate the future application and clinical translation of MNBCs in cardiovascular diseases. Also, such a microneedle‐hidden structure may be integrated with vaginal, respiratory, and urethra catheter balloons for broader applications. Remarkably, the invisible and special design of our MNBCs may enlighten various engineering fields, such as geological prospecting, post‐disaster rescue, narrow space exploration, etc.

## 4 Experimental Section

### Fabrication of the MNBCs

35 w/v% gelatin solution containing 0.1 mg mL^−1^ BP nanosheets and 40 w/v% NAGA solution were set as the gelatin layer material and microneedle layer material, respectively. To fabricate the innermost gelatin coating layer, gelatin solution was first heated to 50 °C and a bare balloon catheter was immersed in the solution. The balloon catheter was then taken out at room temperature for 10 min for solidification. To attach the microneedle layer, the cavities of a microneedle template was first filled with NAGA solution via vacuum treatment for 5 min, with extra solution being removed subsequently. The balloon catheter was pressed on the template to contact parts of the solution‐filled cavities. Ultraviolet (UV) light (365 nm) was applied to cure the NAGA solution that directly contacted the balloon catheter by employing a mask to shield untouched solution from the UV. After that, the balloon catheter rolled along the template and contacted unsolidified solution, and the selective curing was repeated until the surface of the catheter was covered with microneedles completely. To finally add the outermost gelatin coating layer, the above balloon catheter was immersed in the heated gelatin solution and taken out for 10 min again, and the MNBC was obtained.

### Mechanical Strength Testing

A mechanical testing system (Instron) was employed for force measurement. To show the maximum pressure that the microneedles could bear, a 10 × 10 microneedle array made of 40 w/v% NAGA was glued on the bottom fixed platform and the upper movable platform gradually approached it with the speed of 0.1 mm s^−1^. The force measuring began as soon as the movable platform touched the microneedles and ended when it travelled another 0.3 mm. The images of the microneedles before and after pressure were acquired by a stereoscopic microscope (Jiangnan). Besides, to quantify the forces required for blood vessel penetration, a fresh pig aorta tissue was immobilized on the bottom platform and the 10 × 10 microneedle array was glued onto the movable platform. The force detecting began when the microneedles and the tissue contacted and ended until full penetration. The images of the penetration process were taken by the mobile phone (Huawei, P30pro). Additionally, the bright‐field and fluorescence images of the microneedle‐penetrated pig aorta tissue were captured by the fluorescence microscope (Olympus). The fluorescence tomography and reconstruction images of the microneedle‐penetrated rat abdominal aorta tissue were recorded by a laser scanning confocal microscope (Olympus).

### In Vitro Degradation Testing

40 w/v% NAGA hydrogel blocks with a diameter of 1 cm were first fabricated. PBS (pH = 7.4) containing 1 mg mL^−1^ collagenase I was prepared as the buffer solution. The NAGA blocks were immersed in the buffer solution and were oscillated at 100 rpm. At predetermined time points, three NAGA blocks were taken out and their dry weights were recorded.

### Photothermal Responses of MNBCs

To set up the experiment, the MNBC was placed in PBS solution and the NIR emitter (808 nm) was fixed above it. The temperature changes were measured and recorded with the help of infrared thermal images captured by a thermal imager (FLIR, E5xt). To find out the influences of BP concentrations, the BP concentrations varied among 0.1, 0.15, and 0.2 mg mL^−1^, while the NIR power was fixed at 3 W. As for the impacts of NIR powers, the NIR powers changed among 1, 3, and 5 W, while the BP concentration was settled at 0.15 mg mL^−1^.

### NIR‐Triggered Microneedle Exposure and Separation

The three coating layers of MNBCs were dyed red, blue, and green from the exterior to the interior. The colored MNBC was then immersed in PBS and exposed to NIR irradiation. The color changes of PBS as well as the appearances of the MNBC were observed and captured by a mobile phone (Huawei, P30pro). In the intravascular‐simulated experiment, a newly sampled rat abdominal aorta was fixed on the bracket. For full insertion, the MNBC first deflated and shrank. After that, the MNBC inflated and swelled inside the abdominal aorta. Then the NIR light was turned on for 10 min and the abdominal aorta was unfolded after withdrawal of the bare balloon catheter. States of the abdominal aorta and the MNBC were recorded by the mobile phone (Huawei, P30pro). The image of the unfolded abdominal aorta was taken by a fluorescence microscopy (Olympus).

### Subcutaneous Implantation Testing

The mice were anesthetized with isoflurane. For the experiment group, a small incision was created on the mouse dorsal skin and the circular block of MNBC materials (component: 35 w/v% gelatin, 40 w/v% NAGA, and 0.1 mg mL^−1^ BP; diameter: 1 cm) was implanted subcutaneously. For the control group, the mice received only skin incisions without material implantation. The body weights of the mice in both groups were recorded every four days. After 4 weeks, the mice were sacrificed. Their skin tissues around the implanted materials as well as major organs (heart, liver, spleen, lung, and kidney) were sampled. Masson's trichrome staining, H&E staining, and immumohistochemical staining of TNF‐*α* and CD68 were further carried out for histological analysis.

### Establishment and Treatment for Abdominal Aorta Restenosis Rats

The rats were divided into four groups at random: the blank control group (BC‐G), the positive control group (PC‐G), unloaded MNBC treated group (un‐MNBC‐G), and drug‐loaded MNBC treated group (d‐MNBC‐G). BC‐G received sham operation, PC‐G received balloon injury, un‐MNBC‐G received balloon injury together with treatment of MNBC without drug loading, while d‐MNBC‐G received balloon injury together with treatment of drug‐loaded MNBC (rapamycin, 5 mm). Particularly, during the balloon injury, the rats were anesthetized via inhaling isoflurane. The abdominal cavity was surgically opened and the abdominal aorta was exposed. To isolate a zone, microsurgical clamps were used to fix on the proximal and distal abdominal aorta. The adventitia was then dissected at the zone, and heparin was applied to avoid blood coagulation. To create the balloon injury, an inflated balloon catheter (3 mm in diameter) passed through the abdominal aorta for three times. Differently, during the MNBC treatment, the MNBC (2 mm in diameter, 100 µm in microneedle length) was inserted into the abdominal aorta after the former balloon catheter was removed. After inflation, NIR light (808 nm, 5 W) was applied for 60 s to melt the gelatin layer and separate the microneedles. Four weeks after the surgery, the rats were sacrificed and their abdominal aorta tissues were sampled for H&E staining and EVG staining. The maximal intimal thickness, intima to media area ratio, and aortic luminal obliteration were measured and processed from H&E staining images by an image processing software (Image J). Specifically, the aortic luminal obliteration was calculated by the intima area as a percentage of the area inside the internal elastic lamina. The animal experiments have received approval from Animal Investigation Ethics Committee of Nanjing Drum Tower Hospital (2020AE02014).

## Conflict of Interest

The authors declare no conflict of interest.

## Author Contributions

Y.J.Z. designed the idea and provided funding support. X.X.Z. conducted the material experiments and in vitro characterization, analyzed the data, and wrote the manuscript. Y.C. did and supervised the animal experiments. R.L. revised the paper and checked the data.

## Supporting information

Supporting Information

## Data Availability

The data that support the findings of this study are available from the corresponding author upon reasonable request.
