# Supplementary material for: Globefish‐Inspired Balloon Catheter with Intelligent Microneedle Coating for Endovascular Drug Delivery
Source: Adv Sci (Weinh). 2022 Oct 18;9(34):2204497. doi: 10.1002/advs.202204497 (PMC9731713; doi:10.1002/advs.202204497)
Supplement: Supplementary file 1 — Supporting Information [file ADVS-9-2204497-s001.pdf]

ADVANCED SCIENCE
Open Access

Supporting Information

for *Adv. Sci.*, DOI 10.1002/advs.202204497

Globefish-Inspired Balloon Catheter with Intelligent Microneedle Coating for Endovascular Drug Delivery

*Xiaoxuan Zhang, Yi Cheng, Rui Liu and Yuanjin Zhao**

Supporting Information

Globefish-inspired balloon catheter with intelligent microneedle coating for endovascular drug delivery

*Xiaoxuan Zhang, Yi Cheng, Rui Liu, Yuanjin Zhao**

Supporting Methods

Materials: Gelatin (from porcine skin, ~300 g) and N,N'-Methylenebis(acrylamide) (Bis) were purchased from Sigma-Aldrich. N-acryloyl glycinamide (NAGA) was provided by Alfachem Co., Ltd. (Zhengzhou, China). BP nanosheets were from XFNANO Co., Ltd. (Nanjing, China). FluoSpheres^{TM} carboxylate-modified (0.2 µm, 365/415, 505/515, and 580/605) were bought from Invitrogen. Nile Red was offered by Aladdin (China). Pigment inks (red, green, and blue) were purchased from Deli Co., Ltd. (China). CCK-8 kits (Cat. NO: KGA317) and cell live/dead staining kits (Cat. NO: KGA214) were provided by KeyGEN BioTECH Co., Ltd. (Nanjing, China). Fetal bovine serum (FBS), penicillin-streptomycin double antibiotics (PS), Dulbecco's Modified Eagle Medium (DMEM), phosphate buffer saline (PBS, pH = 7.4) were bought from Gibco. Balloon catheters were from Nanjing Tower Drum Hospital and microneedle templates were from WISECARE Co., Ltd. (Taizhou, China). Deionized water (dH_2O) with a resistivity of 18.2 MΩ cm^{-1} was generated by a Milli-Q water purification system (Millipore). All chemical reagents were of analytical grade and used as received.

Cells, animal tissues, and animals: Human umbilical vein endothelial cells (HUVECs) were obtained from Nanjing Drum Tower Hospital and were cultivated in DMEM with 10 v/v% FBS and 1 v/v% PS at 37°C, 5% CO_2. 20-25 g male BALB/c mice and 300-350 g male Wistar rats were provided by Nanjing Drum Tower Hospital. Animals were treated in strict accordance with the Beijing Administration Rule of Animals in China and have received approval from Animal Investigation Ethics Committee of Nanjing Drum Tower Hospital. Fresh pig aorta tissues were

bought from the Jinxinghe supermarket in Nanjing. Rat abdominal aorta tissues and rat blood were collected from newly sacrificed or anesthetic rats.

Cyto-compatibility testing: Leaching liquors of the MNBC materials were first prepared by immersing different materials (1 cm × 1 cm × 1 cm blocks) in 10 mL of the culture medium for 24 h, respectively. HUVECs were randomly divided into five groups: in control group, the cells were cultured in 1mL traditional culture medium; in NAGA group, the cells were in 1 mL leaching liquor of 40 w/v% NAGA hydrogel; in GEL group, they were in 1 mL leaching liquor of 35 w/v% gelatin hydrogel; in GEL+BP group, they were in 1 mL leaching liquor of 35 w/v% gelatin hydrogel that contained 0.1 mg/mL BP; in NAGA+GEL+BP group, they were in 1 mL leaching liquor of gelatin (35 w/v%) / NAGA (40 w/v%) mixed hydrogel that contained 0.1 mg/mL BP. After co-culture for 3 d, live HUVECs were stained with Calcein, AM, and dead cells were stained with PI. Cell fluorescence images were taken by the fluorescence microscope (Olympus). CCK-8 assays were performed on day 1, 2, and 3. For each day, the original culture medium was discarded and 500 µL fresh traditional culture medium containing 50 µL CCK-8 was replaced. After 3 h, their OD value at 450 nm was read out by a microplate reader (Thermo Scientific).

Hemolysis testing: 2% rat blood cell solution was first prepared by dispersing 1 mL precipitations of fresh rat whole blood in 49 mL PBS. Leaching liquors of the MNBC materials were obtained by immersing different materials (1 cm × 1 cm × 1 cm blocks) in 10 mL PBS for 24 h. The grouping mode was the same as the cyto-compatibility testing. For different groups, the blood cell solution and the water, PBS, or leaching liquor were mixed at the proportion of 1:1. The mixture was then incubated at 37℃ for 4 h. After centrifuging at 3500 rpm for 5 min, the OD value of the supernatant at 570 nm was recorded by the microplate reader (Thermo Scientific). The hemolysis rates of the materials could be calculated based on the equation:

$$\mathrm{Hemolysis}_{\mathrm{material}}(\%) = (\mathrm{OD}_{\mathrm{material}} - \mathrm{OD}_{\mathrm{PBS}})/(\mathrm{OD}_{\mathrm{dH2O}} - \mathrm{OD}_{\mathrm{PBS}})$$

Ex vivo drug release: 0.5 mg/mL Nile Red was loaded in the microneedles consisting of 40 w/v% NAGA. The microneedles were immersed in DMSO solution and incubated at 37℃. 100 µL of DMSO solution was pipetted at specific time intervals and the OD value at 530 nm was read using the microplate reader (Thermo Scientific). At the same time, 100 µL of fresh DMSO

solution was supplemented. Based on the OD values and the standard concentration curve of Nile Red, cumulative release (CR) of Nile Red from the microneedles could be calculated. It should be mentioned that the crosslinker, Bis, was introduced to adjust the drug release profile of the microneedles. For the none group, no Bis was added to the NAGA hydrogel. For the 200:1 group, the mass ratio of NAGA and Bis was 200:1. For the 50:1 group, the mass ratio of NAGA and Bis was 50:1.

Statistical analysis: Data were normalized with the control group as the standard and were presented in the form of mean ± SD. Sample size (n) for each statistical analysis was provided in the figure legends. Image J and OriginPro 8.5 were the software used for statistical analysis.

Supporting Figures

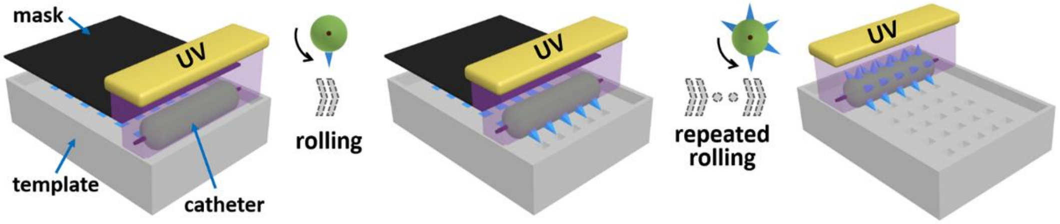

Figure S1. Schematics showing the process of assembling microneedles on the gelatin coated balloon catheter. This process is achieved by repeated rolling the catheter and mask-assisted UV irradiation.

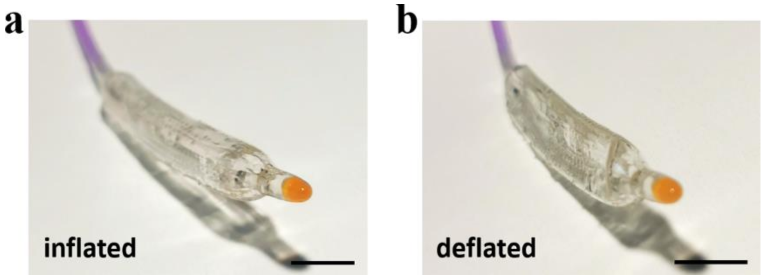

Figure S2. Photos showing the three-layered coating conforming to the balloon catheter as it inflates (**a**) and deflates (**b**). Scale bars: 5 mm.

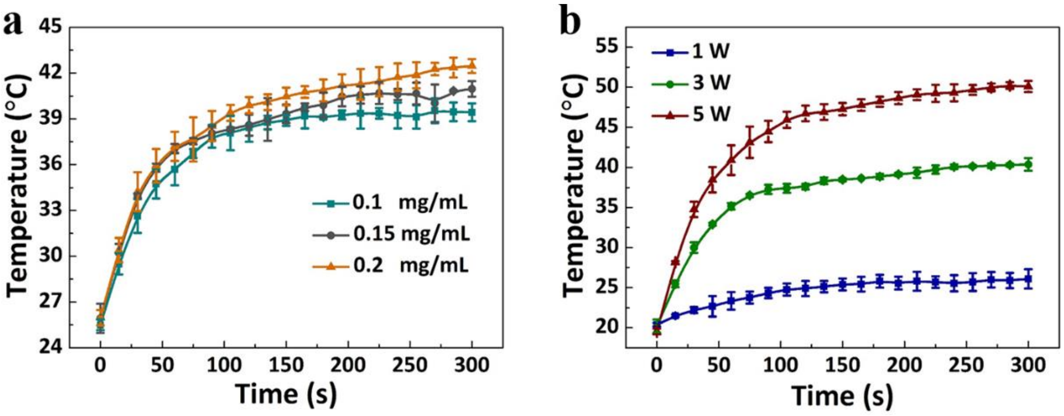

Figure S3. Factors that affect the photothermal conversion. (**a**) Heating curves of the gelatin layer when the BP concentration is 0.1 mg/mL, 0.15 mg/mL, and 0.2 mg/mL, respectively (n = 3 for each group). (**b**) Heating curves when the NIR power is 1 W, 3 W, and 5 W, respectively (n = 3 for each group).

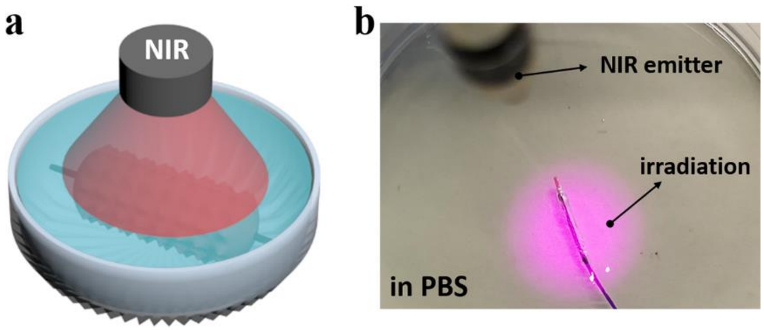

Figure S4. Experimental setup of the NIR-triggered microneedle exposure and separation in PBS. (**a**) Schematic illustration. (**b**) Digital image.

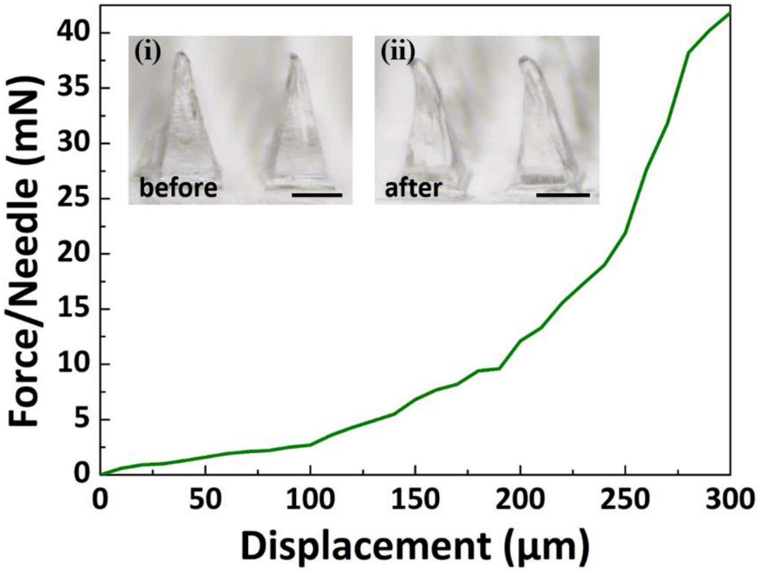

Figure S5. Force-displacement curve showing the pressure that each microneedle can tolerate. Inset: optical image of the microneedles before (**i**) and after (**ii**) pressure. Scale bars: 300 μm.

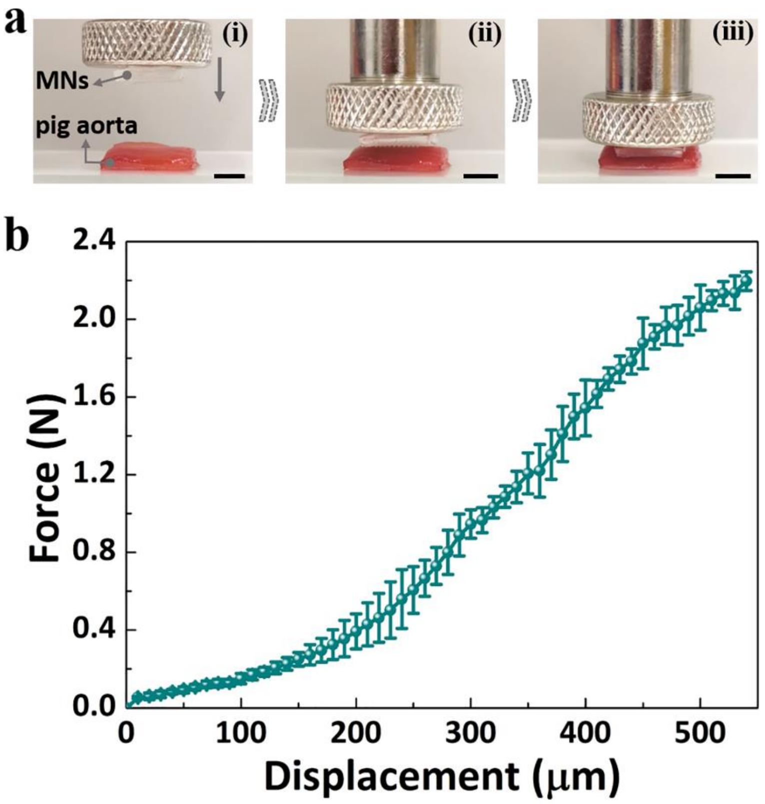

Figure S6. Forces that microneedles impose on the pig aorta tissue during penetration. (**a**) Photos showing the penetration process. (**b**) Force-displacement curve during the entire penetration (n = 3 for each group). Scale bars: 4 mm.

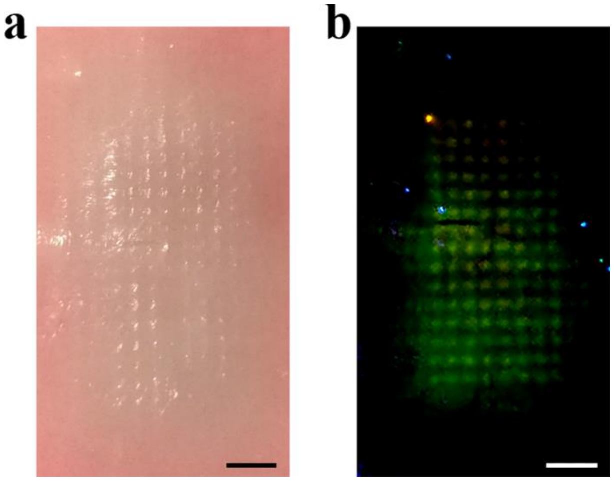

Figure S7. Optical images of the pig aorta tissue after microneedle penetration. (**a**) Bright field image. (**b**) Corresponding fluorescence image. The microneedles are dyed with green fluorescence. Scale bars: 2 mm.

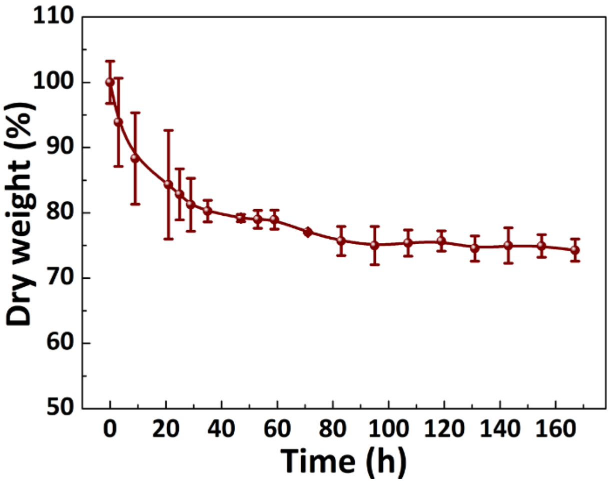

Figure S8. Dry weight changes of NAGA hydrogel blocks immersed in buffer solutions (n = 3 at each time point and the normalization was based on the data of 0 h).

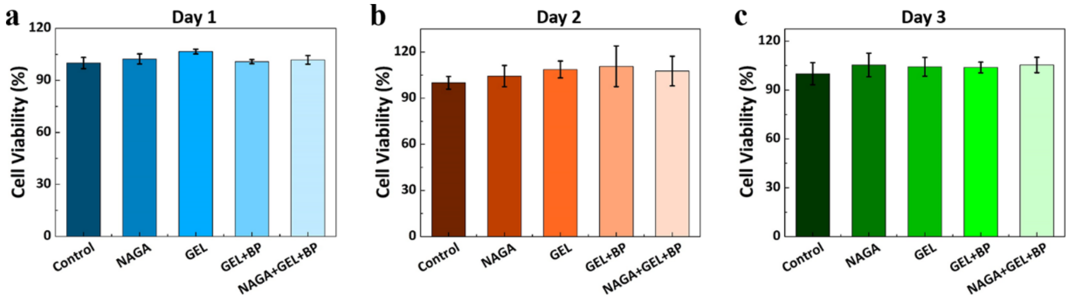

Figure S9. Viability of HUVECs in traditional culture medium (control) and leaching liquors of NAGA hydrogel (NAGA), gelatin hydrogel (GEL), BP-containing gelatin hydrogel (GEL+BP), and BP-containing NAGA-gelatin mixed hydrogel (NAGA+GEL+BP) on day 1 (**a**), day 2 (**b**), and day 3 (**c**), respectively (n = 8 for each group and data normalization was based on the control group).

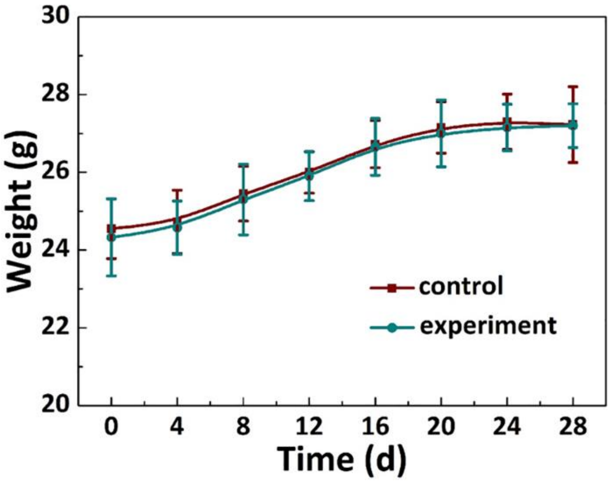

Figure S10. Body weight changes of mice without (control group) and with (experiment group) material implantation (n = 4 for each group).

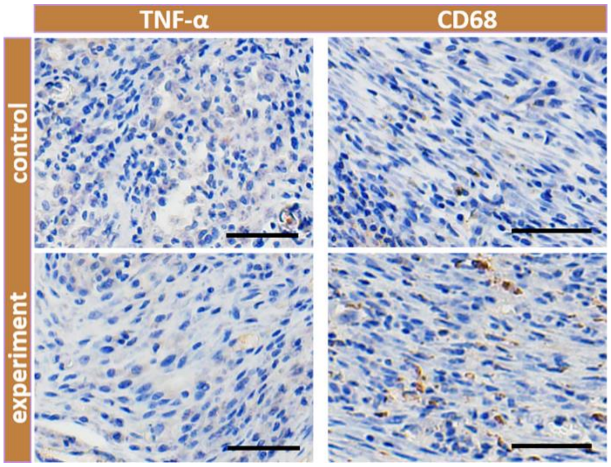

Figure S11. Immumohistochemical staining of TNF-α and CD68 of mouse skins without (control group) and with (experiment group) material implantation on day 28. Scale bars: 50 μm.

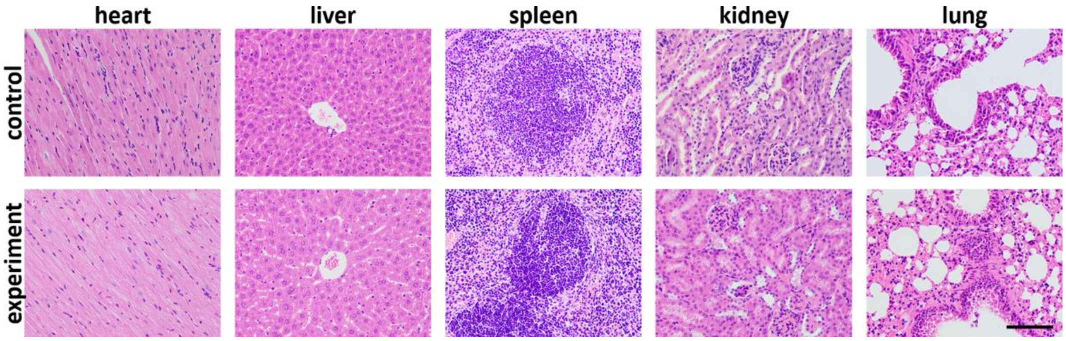

Figure S12. H&E staining of heart, liver, spleen, kidney, and lung of mice without (control group) and with (experiment group) material implantation, respectively. Scale bar: 100 μm.

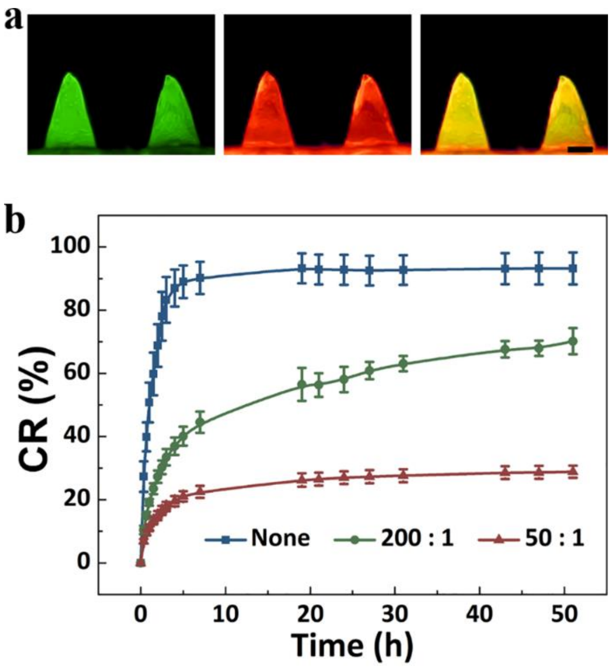

Figure S13. Drug loading and release of the microneedles of MNBCs. (**a**) Fluorescence images showing Nile Red distribution inside the microneedles. The microneedles are dyed with green fluorescence. (**b**) Cumulative release (CR) rates of Nile Red from microneedles composed of pure NAGA (none), NAGA-crosslinker mixture with the ratios of 200:1 and 50:1, respectively (n = 4 for each group). Scale bar: 150 μm.

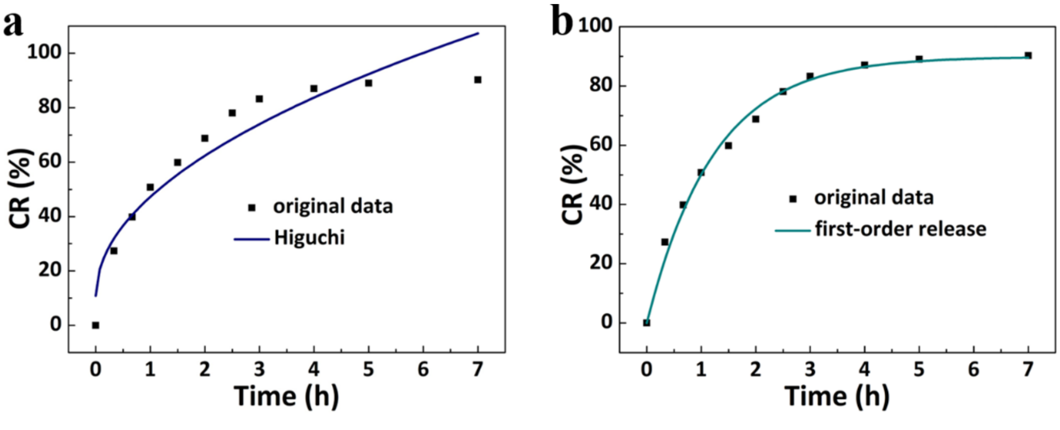

Figure S14. Fitting curves of drug release from pure NAGA-composed microneedles before reaching plateau. (**a**) Fitting curve using Higuchi equation. (**b**) Fitting curve using first-order release equation.

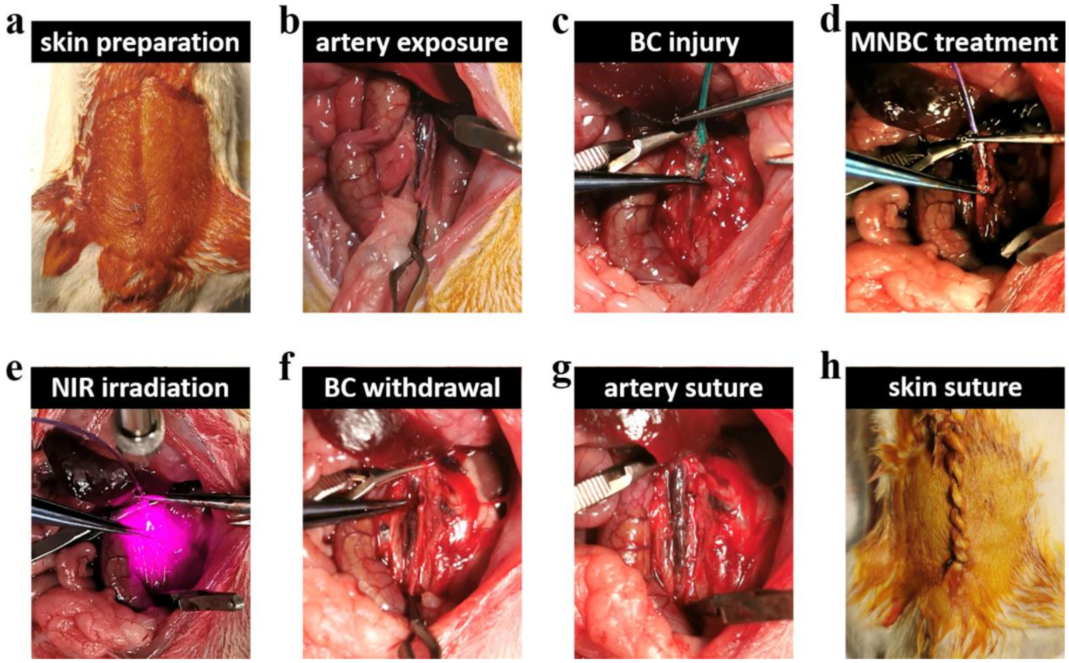

Figure S15. The procedures of model establishment and treatment. (**a**) Preparation of the rat abdomen skin. (**b**) Exposure of the rat abdominal aorta. (**c**) Abdominal aorta injury induced by balloon catheter. (**d**) Placement of the drug-loaded MNBC for treating. (**e**) NIR irradiation for microneedle maintenance and drug release. (**f**) Removal of the bare balloon catheter. (**g**) Suturing the abdominal aorta. (**h**) Suturing the abdomen skin.

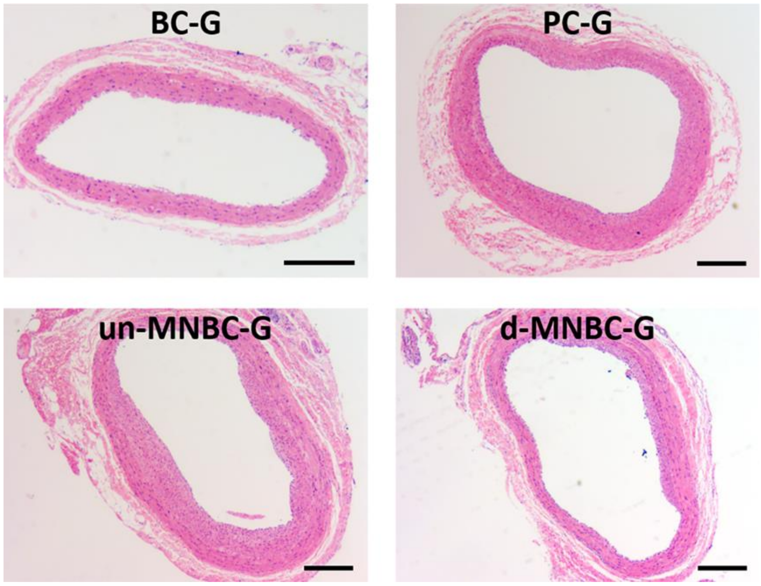

Figure S16. H&E staining of the cross sections of the abdominal aorta tissues from the four groups. Scale bars: 250 µm.
